# 超高效液相色谱-串联质谱法快速测定血浆中维奈克拉药物

**DOI:** 10.3724/SP.J.1123.2025.06002

**Published:** 2026-03-08

**Authors:** Ying ZHU, Xiaoli MA, Songlin YU, Ling QIU

**Affiliations:** 中国医学科学院北京协和医学院，北京协和医院检验科，北京 100730; Department of Laboratory Medicine，Peking Union Medical College Hospital，Peking Union Medical College & Chinese Academy of Medical Science，Beijing 100730，China

**Keywords:** 超高效液相色谱-串联质谱, 维奈克拉, 治疗药物监测, 血浆, ultra performance liquid chromatography-tandem mass spectrometry （UPLC-MS/MS）, venetoclax, therapeutic drug monitoring, plasma

## Abstract

维奈克拉是一种B细胞淋巴瘤因子-2抑制剂，对急性髓系白血病和慢性淋巴细胞白血病等血液系统恶性肿瘤的临床治疗具有重要作用。维奈克拉呈现显著个体间药代动力学差异，谷浓度和治疗反应显著相关，峰浓度超过警戒浓度又会引发不良反应，因此监测维奈克拉血药浓度变化至关重要。本研究建立了一种快速、灵敏、可靠的超高效液相色谱-串联质谱法（UPLC-MS/MS）用于定量测定血浆中的维奈克拉药物浓度。该方法采用电喷雾电离，正离子模式下多反应监测（MRM）检测维奈克拉及其同位素内标维奈克拉-d_8_。前处理采用甲醇进行蛋白沉淀。液相分离使用C18反相色谱柱，用乙腈和0.1%甲酸水溶液作为流动相进行梯度洗脱，维奈克拉保留时间为1.95 min。方法学验证结果显示维奈克拉线性范围为50~10 000 ng/mL，*r* ^2^>0.999，方法特异性良好，批内精密度和批间精密度分别为1.8%~4.5%和2.7%~6.1%，回收率为100.3%~102.9%，基质效应为88.0%~111.0%，进样最高浓度维奈克拉（10 000 ng/mL）后在空白样本中的残留小于线性范围最低浓度峰面积的20%，满足临床要求。将该方法应用于3例急性髓系白血病患者维奈克拉的治疗药物监测，3例患者血浆维奈克拉的最高峰浓度为5 357.9 ng/mL，最低谷浓度为438.2 ng/mL。上述研究结果表明该方法能够准确、高效地定量维奈克拉血药浓度，有助于解决白血病患者的维奈克拉治疗药物浓度监测问题，指导临床个性化治疗。

## Abstract (en)

Leukemia is a malignant tumor of the hematological system characterized by the uncontrolled proliferation of abnormal hematopoietic cells in the bone marrow. It often presents with anemia， bleeding tendency， infection risk and organ invasion. These clinical symptoms bring severe survival risks to patients. Although traditional chemotherapy regimens are effective in the treatment of some hematological malignancies， their efficacy is limited for elderly patients， those with high-risk genetic characteristics or comorbidities. In recent years， targeted drugs have revolutionized the treatment of leukemia. By selectively inducing tumor cell apoptosis， they have significantly improved the remission rate and survival prognosis of patients with multiple leukemia subtypes. Venetoclax is a B-cell lymphoma 2 （BCL-2） inhibitor and plays an important role in the clinical treatment of hematological malignancies， such as acute myeloid leukemia and chronic lymphocytic leukemia. In addition， it also shows potential efficacy in other hematological malignancies such as multiple myeloma and mantle cell lymphoma. Although the efficacy of venetoclax is remarkable， the individual differences in blood drug concentration are significant due to factors such as drug interactions， polymorphisms of metabolic enzymes， and liver and kidney function. Venetoclax exhibits significant inter-individual pharmacokinetic differences， the trough concentration is significantly correlated with the treatment response， and if the peak concentration exceeds the warning concentration， adverse reactions will be triggered. Clinical trials have reported a variety of adverse events associated with venetoclax， including neutropenia， tumor lysis syndrome， thrombocytopenia， infection， anemia， diarrhea， nausea， upper respiratory tract infection， cough and musculoskeletal pain. Therefore， to minimize the risk of adverse events in the clinical use of venetoclax as much as possible， it is necessary to reasonably guide its clinical dosage. Therapeutic drug monitoring can optimize individual dosing regimens by measuring the steady-state concentration in patients’ blood. This research aims to establish a rapid， sensitive and reliable ultra performance liquid chromatography-tandem mass spectrometry （UPLC-MS/MS） method. This method is used for the quantitative determination of venetoclax in plasma， and its performance was validated. This method employs an electrospray ionization and multiple reaction monitoring （MRM） in the positive ion mode to detect venetoclax and its isotope internal standard venetoclax-d_8_. Methanol was used for protein precipitation， C18 reversed-phase chromatographic column was used for liquid phase separation. Gradient elution was performed using acetonitrile and 0.1% formic acid aqueous solution as the mobile phases. The flow rate was 0.4 mL/min， the run time was 3.5 min， and the retention time of venetoclax was 1.95 min. The analysis time is short， facilitationg the rapid determination of clinical samples. Dosage escalation is commonly adopted in the treatment of venetoclax. In this study， a pretreatment approach involving extraction followed by dilution was used. This methodincreased the upper limit of quantification and expanded the linear range. The linear range of venetoclax was 50–10 000 ng/mL， *r* ^2^>0.999. The method had good specificity. The intra-run precision and inter-run precision were 1.8%–4.5% and 2.7%–6.1%， respectively， the recoveries were 100.3%–102.9%， the matrix effects ranged from 88.0% to 111.0%， and the carryover was less than 20% of the minimum concentration of linear range. This method was applied to the therapeutic drug monitoring of venetoclax in acute myeloid leukemia （AML） patients， and the peak and trough concentrations of venetoclax were obtained for different patients to monitor their blood drug concentration. The above research results indicate that this method can accurately and robustly quantify venetoclax in human plasma， which helps address the drug monitoring needs of leukemia patients receiving venetoclax treatment and guide clinical personalizd therapy.

白血病是以骨髓中异常造血细胞的失控增殖为特征的血液系统恶性肿瘤，根据细胞起源或分化阶段不同，可分为急性髓系白血病、慢性淋巴细胞白血病、骨髓增生性肿瘤和骨髓增生异常综合征等多种亚型^［1，2］^。白血病常表现出贫血、出血倾向、感染风险及器官浸润等，这些临床症状给患者带来严峻的生存风险。传统化疗方案虽对部分血液系统恶性肿瘤治疗有效，但对老年患者、存在高危遗传学特征或合并症等人群的疗效有限，不仅复发率高而且具有严重治疗毒性^［3］^。近年来靶向药物革新了白血病的治疗，通过选择性抑制诱导肿瘤细胞凋亡，显著提升了多种白血病亚型患者的缓解率与生存预后^［4］^。

维奈克拉（venetoclax）是一种高选择性B细胞淋巴瘤因子-2（BCL-2）抑制剂，通过靶向抑制抗凋亡蛋白BCL-2，解除肿瘤细胞对凋亡通路的抑制，从而诱导肿瘤细胞程序性死亡^［5］^。目前美国食品药品监督管理局（FDA）批准的维奈克拉适应证包括急性髓系白血病和慢性淋巴细胞白血病/小淋巴细胞淋巴瘤等^［6］^。对于老年或无法耐受强化疗的急性髓系白血病人群，维奈克拉联合阿扎胞苷等去甲基化药物可显著提升缓解率，如强化化疗在老年急性髓系白血病患者中完全缓解率约为40%~60%，而维奈克拉联合去甲基化药物完全缓解率高达74%^［7］^。而在慢性淋巴细胞白血病/小淋巴细胞淋巴瘤的治疗中，维奈克拉单药治疗和联合奥妥珠单抗或依鲁替尼等方案也表现出较好的治疗效果^［8］^。此外有研究还探索了维奈克拉在多发性骨髓瘤、套细胞淋巴瘤等其他血液肿瘤中的疗效，扩展了其潜在临床应用范围^［9］^。

尽管维奈克拉的疗效显著，但受患者药物相互作用、代谢酶多态性和肝肾功能等因素影响，血药浓度个体差异显著^［10，11］^，血药浓度不足可能导致治疗失败，而浓度过高则增加安全性风险。有研究报道了维奈克拉可引起多种不良反应，包括中性粒细胞减少、肿瘤溶解综合征、血小板减少症、感染、贫血、腹泻、恶心、上呼吸道感染、咳嗽和肌肉骨骼疼痛等^［6］^。因此，为尽可能降低维奈克拉临床使用的不良事件风险，需合理指导其临床使用剂量。治疗药物监测通过测定药物在患者血液中的稳态浓度，可优化个体给药方案，用于确保合适药物暴露剂量和限制剂量相关的毒性。

液相色谱-串联质谱技术（LC-MS/MS）具有高灵敏度、高选择性优势，已成为临床检验常用的定量检测方法^［12］^。针对维奈克拉药物浓度检测，已有研究建立了基于LC-MS/MS的定量检测方法，Alnasser等^［13］^采用固相萃取前处理和LC-MS/MS方法测定了大鼠血浆中维奈克拉的含量，方法定量范围为5~1 000 ng/mL，但前处理步骤烦琐且动态范围窄。在临床使用时，患者通常采取用药剂量“爬坡”方案，例如在急性髓系白血病治疗中，维奈克拉常规采用第一天100 mg，第二天200 mg，第三天400 mg，之后每天400 mg继续治疗，每疗程28天，动态检测范围需求较宽。Yang等^［14］^开发了一种有机溶剂沉淀蛋白LC-MS/MS方法检测人血浆中的维奈克拉浓度，测得的患者最大的维奈克拉峰浓度为7 189.0 ng/mL，建立的方法线性范围为25~8 000 ng/mL，但患者血药峰浓度与定量上限浓度仍较接近，因此需建立覆盖更高定量上限的方法，以满足可能的高血药浓度临床需求。

本研究旨在建立一种宽动态范围、快速、灵敏、可靠的超高效液相色谱-串联质谱方法用于准确定量人血浆中维奈克拉的药物浓度，并进行方法学验证。将建立的方法应用于临床急性髓系白血病患者的治疗药物监测，探究维奈克拉血药浓度的个体差异。

## 1 实验部分

### 1.1 仪器、试剂与材料

#### 1.1.1 仪器与试剂

ACQUITY UPLC I-Class/Xevo TQ-S超高效液相色谱-三重四极杆质谱仪（美国Waters公司），Sorvall Legend Micro17R微量离心机（美国Thermo Scientific公司），MIX-25混匀仪（杭州米欧仪器有限公司）。

维奈克拉标准品（纯度>98.00%）购自美国GlpBio公司，维奈克拉-d_8_标准品（纯度99.76%）购自美国MedChemExpress公司，甲醇、乙腈（色谱纯）购自美国Thermo Scientific公司，甲酸（质谱纯）购自霍尼韦尔贸易上海有限公司。

#### 1.1.2 血浆样本

收集2024年3月至8月在北京协和医院进行治疗的3例急性髓系白血病患者的6个血浆样本，男性2例，女性1例，年龄分别为29、59和48岁，分析前血浆样本存放于-80 ℃冰箱。本研究经北京协和医院医学伦理委员会审查并通过（批件号：I-PJ240722）。

### 1.2 标准品溶液和内标溶液的配制

以二甲基亚砜为溶剂，配制质量浓度为2.0 mg/mL的维奈克拉溶液作为储备液，配制质量浓度为1.0 mg/mL的维奈克拉-d_8_溶液作为内标储备液，于-80 ℃储存。采用甲醇溶液进一步稀释得到20 ng/mL维奈克拉-d_8_内标溶液；并采用甲醇溶液配制系列标准曲线溶液，质量浓度分别为0.5、1、2、5、10、20、50和100 μg/mL。

### 1.3 样本前处理

吸取100 μL患者血浆样本，加入1 mL甲醇，涡旋10 min后离心（13 000 r/min，10 min）；取20 μL上清液加入380 μL含20 ng/mL维奈克拉-d_8_内标的甲醇溶液，涡旋混匀后转移100 μL至96孔板，进样1 μL。

### 1.4 色谱-质谱分析条件

采用Waters公司ACQUITY UPLC BEH C18色谱柱（50 mm×2.1 mm， 1.7 µm），流动相A为0.1%甲酸水溶液，流动相B为乙腈溶液，流动相流速为0.4 mL/min，梯度洗脱程序如下：0~0.5 min，85%A；0.5~2.0 min，85%A~5%A；2.0~2.5 min，5%A；2.5~3.5 min，85%A。柱温为35 ℃，样本室温度为10 ℃。

质谱条件为在正离子模式下，基于电喷雾离子化的多反应监测模式进行分析，参数如表1所示。毛细管电压为2.50 kV，脱溶剂气温度为550 ℃，脱溶剂气气流为1 100 L/h，锥孔气流为150 L/h，雾化气气流为700 kPa。

**表1:** 维奈克拉和维奈克拉-d_8_的质谱参数

Compound	Precursor ion （*m/z*）	Product ion （*m/z*）	Cone voltage/V	Collision energy/eV
Venetoclax	868.3	321.0	30	40
		636.2^*^	30	25
Venetoclax-d_8_	876.3	329.1	42	36
		644.2^*^	42	26

* Quantitative ion.

## 2 结果与讨论

### 2.1 方法建立与优化

实验采用注射泵直接进样的方式将500 ng/mL维奈克拉和500 ng/mL维奈克拉-d_8_标准溶液依次注入质谱仪中，在ESI^+^模式下进行母离子扫描，选择维奈克拉和维奈克拉-d_8_的［M+H］^+^作为母离子，对母离子进行子离子扫描，选择质谱丰度响应值高且信号稳定的碎片离子作为子离子。接着对这些离子对的锥孔电压和碰撞能量进行优化，以获得合适的MRM信号强度，结果如表1所示。

样本前处理策略采用甲醇沉淀蛋白，因为维奈克拉血药浓度较高，样本检测时离子强度高易导致仪器过饱和，以及出现残留效应。为避免这些问题，我们采用血浆样本和甲醇的体积比为1∶10的比例沉淀蛋白，进一步取上清，采用上清和内标工作溶液体积比为1∶19的比例进行再次稀释。

### 2.2 方法学考察

#### 2.2.1 特异性

血浆样本加入维奈克拉和维奈克拉-d_8_前后色谱图如图1所示。在加入维奈克拉和维奈克拉-d_8_前，血浆样本在预期保留时间的位置未观察到明显的色谱峰（图1a）。当加入维奈克拉和维奈克拉-d_8_后，在1.95 min左右观测到明显的色谱峰（图1b），并且提取离子流图显示在维奈克拉和维奈克拉-d_8_保留时间前后均未见明显的干扰峰，说明该方法对维奈克拉和内标维奈克拉-d_8_的测定特异性良好。

**图1:**
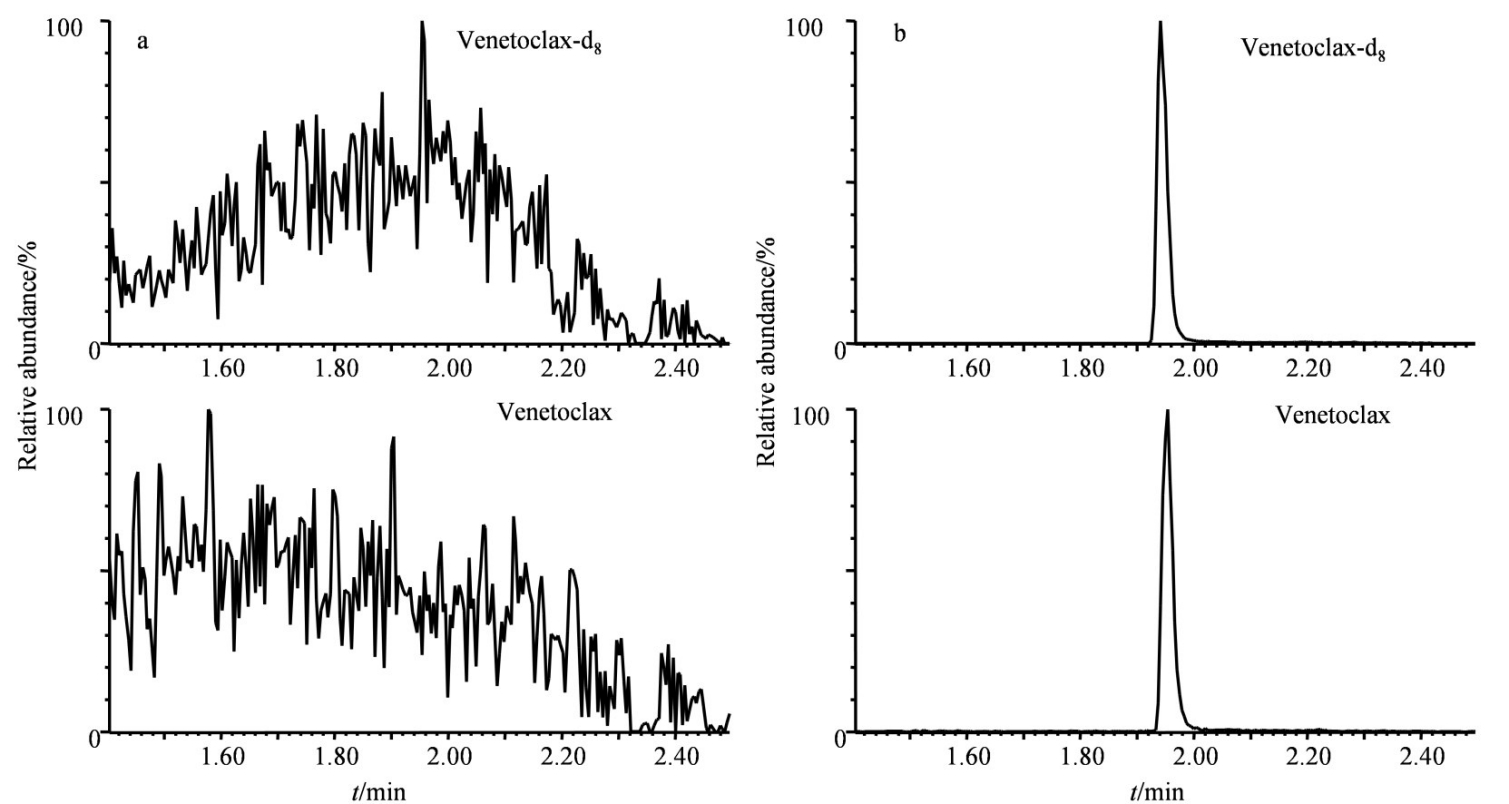
血浆样本加入维奈克拉和维奈克拉-d_8_（a）前、（b）后的提取离子流色谱图

#### 2.2.2 线性

以维奈克拉及内标维奈克拉-d_8_峰面积之比作纵坐标（*Y*），以血浆维奈克拉质量浓度为横坐标（*X，*ng/mL），用加权（*W*=1/*X*）最小二乘法进行回归和线性拟合，得到标准曲线。结合临床实际应用需求确定定量限（LOQ）和检出限（LOD），LOQ需通过20次进样，满足最小信噪比（*S/N*）>10，变异系数（CV）<20%，LOD需通过20次进样，*S/N*>3，CV<20%。血浆中维奈克拉在50~10 000 ng/mL范围内，回归方程*Y*=0.000 316 8*X*+0.001 935，相关系数*r* ^2^>0.999，表明方法线性良好，定量限为50 ng/mL，检出限为10 ng/mL。Yang等^［13］^报道中国急性髓系白血病患者血浆中维奈克拉质量浓度范围为74.2~7 189.0 ng/mL。本研究的校准曲线可以覆盖维奈克拉常规血药浓度，且对可能的高浓度维奈克拉样本仍线性良好。因此本方法适用于临床患者血浆中维奈克拉的浓度测定。

#### 2.2.3 精密度

采用同一天分别重复测定5次低、中、高水平质控样本来评估批内精密度，以不同天5次批内精密度实验来评估批间精密度。维奈克拉的批内精密度为1.8%~4.5%，批间精密度为2.7%~6.1%，总精密度为2.9%~6.4%，均小于15%，表明该方法精密度良好，符合要求。

#### 2.2.4 回收率和基质效应

取3个不同浓度的维奈克拉标准溶液分别添加到空白血浆中，配制成200、1 000、2 000 ng/mL的加标样本，然后将每个浓度各3份加标样本处理后进样检测，将实测值与理论值相比较计算加标回收率。3个水平下加标血浆样本中维奈克拉的提取回收率为100.3%~102.9%，RSD≤5%。

采用提取后加标法评价基质效应，将2 000、10 000、20 000 ng/mL 3个不同水平的标准溶液及内标分别加标到6份不同的空白血浆提取液和水溶剂的提取液中，比较分析物（维奈克拉/内标）在血浆中的相对响应与在空白溶剂中的相对响应。结果表明，维奈克拉在3个水平下的基质效应分别为106.2%±4.6%、93.7%±3.1%、94.0%±4.5%，结果符合国际人用药品注册技术协调会（ICH）M10^［15］^要求。

#### 2.2.5 携带污染

进样线性范围最高浓度点的血浆样本后，连续3次进样空白血浆样本来评估残留效应，通过计算得到维奈克拉残留为3.80%，小于线性范围最低浓度点峰面积20%，维奈克拉-d_8_残留为0.05%，小于内标维奈克拉-d_8_峰面积的5%，均符合要求。

### 2.3 与已发表检测方法比较

表2列出了已发表的液相色谱-质谱检测生物样品中维奈克拉药物浓度的方法，与既往文献报道相比，本方法动态范围宽，适合浓度波动大的维奈克拉药物浓度监测，且保留时间短，并具有较高的分析通量。

**表 2:** 已发表的测定维奈克拉药物的LC-MS/MS方法

Sample	Mobile phase	Column	Acquisition time/min	Linear range/ （ng/mL）	Publication year	Ref.
Rat plasma	0.1% formic acid in water -acetonitrile （50∶50， *V/V*， pH 3.2）	Agilent Eclipse plus C18 column （100 mm×2.1 mm， 3.5 µm）	3.0	5-1000	2023	［^13^］
Humuan plasma	0.1% formic acid in water and acetonitrile	ACQUITY UPLC BEH C18 column （100 mm×2.1 mm， 1.7 μm）	4.0	25-8000	2022	［^14^］
Rat plasma	0.1% formic acid in water -acetonitrile （50∶50， *V/V*， pH 3.2）	Eclipse plus C18 column （100 mm×2.1 mm， 1.8 μm）	2.5	5-1000	2025	［^16^］
Human cerebrospinal fluid， bone marrow， and plasma	NA	NA	NA	30-8000	2024	［^17^］
rat plasma	10 mmol/L ammonium formate and 0.1% formic acid acetonitrile and methanol	Phenomenex Kinetex C18 （150 mm×2.1 mm， 2.6 µm）	6.0	5-500	2021	［^18^］
Human plasma and cerebrospinal fluid	10 mmol/L ammonium acetate containing 0.1% formic acid and acetonitrile	ACQUITY UPLC HSS T3 column （50 mm×2.1 mm， 1.8 μm）	5.0	20-5000	2023	［^19^］
Human plasma	0.1% formic acid in water and acetonitrile	ACQUITY UPLC BEH C18 column （50 mm×2.1 mm， 1.7 μm）	4.0	125-8000	2023	［^20^］

NA： not available.

### 2.4 临床应用

采用建立的UPLC-MS/MS方法，对接受维奈克拉治疗的急性髓系白血病患者血浆样本进行药物浓度检测。3例患者的峰浓度（*C*_max_）和谷浓度（*C*_min_）检测结果如表3所示。结果表明，不同患者峰浓度和谷浓度个体差异较大，3例患者的维奈克拉峰浓度随着年龄增加而升高。有文献报道基于62例中国急性髓系白血病患者检测的维奈克拉峰浓度范围为515.4~7 189.0 ng/mL，谷浓度范围为74.2~3 257.0 ng/mL^［14］^，患者一和患者三的药物浓度与之相符，患者二*C*_min_（4 342.8 ng/mL）高于文献谷浓度范围，该患者联合使用了维奈克拉和泊沙康唑进行治疗，且年龄较大，维奈克拉主要通过细胞色素P450 3A酶（CYP3A）代谢，泊沙康唑是强效的CYP3A抑制剂^［21］^，联合使用时使维奈克拉代谢受阻，血药浓度升高，可能是该患者谷浓度较高的原因。本研究结果表明进行治疗药物监测对于维持个体有效药物浓度具有重要意义。

**表 3:** 3例患者血浆中维奈克拉的峰浓度和谷浓度

Patient No.	Gender	Age/year	*C*_max_/（ng/mL）	*C*_min_/（ng/mL）
1	male	29	2860.0	1284.3
2	male	59	5357.9	4342.8
3	female	48	3217.4	438.2

## 3 结论

维奈克拉是一种重要的白血病靶向治疗药物，能够高选择性抑制BCL-2，其药代动力学具有显著的个体差异，影响药效和安全性。本研究建立了一种基于UPLC-MS/MS技术定量检测临床患者血浆样本维奈克拉药物浓度的方法，分析耗时短，有利于临床样本的快速测定。本研究方法线性范围宽，以适用于维奈克拉治疗常采取的用药剂量“爬坡”策略，方法学验证结果表明线性、特异性、批内和批间精密度、回收率、基质效应和残留较好，满足临床样本分析要求。最后将该方法应用于急性髓系白血病患者维奈克拉治疗药物监测，指导了患者个性化治疗。本研究建立的UPLC-MS/MS方法操作简单，检测快速，能够准确、高效地定量维奈克拉血药浓度，不仅可辅助制定个体化给药方案以平衡疗效与毒性，还为药效学研究和耐药机制探索提供技术支撑。
